# An RFID-Based Smart Structure for the Supply Chain: Resilient Scanning Proofs and Ownership Transfer with Positive Secrecy Capacity Channels †

**DOI:** 10.3390/s17071562

**Published:** 2017-07-04

**Authors:** Mike Burmester, Jorge Munilla, Andrés Ortiz, Pino Caballero-Gil

**Affiliations:** 1 Department of Computer Science, Florida State University, Tallahassee, FL 32304, USA; burmester@cs.fsu.edu; 2 Escuela Técnica Superior de Ingenieros de Telecomunicación, Universidad de Málaga, 29070 Málaga, Spain; aortiz@ic.uma.es; 3 Facultad de Ciencias, Departamento de Ingeniería de Informática y de Sistemas, Universidad de La Laguna, 38271 Tenerife, Spain; pcaballe@ull.es

**Keywords:** RFID, grouping proof, ownership transfer, supply chain, secrecy capacity

## Abstract

The National Strategy for Global Supply Chain Security published in 2012 by the White House identifies two primary goals for strengthening global supply chains: first, to promote the efficient and secure movement of goods, and second to foster a resilient supply chain. The Internet of Things (IoT), and in particular Radio Frequency Identification (RFID) technology, can be used to realize these goals. For product identification, tracking and real-time awareness, RFID tags are attached to goods. As tagged goods move along the supply chain from the suppliers to the manufacturers, and then on to the retailers until eventually they reach the customers, two major security challenges can be identified: (I) to protect the shipment of goods that are controlled by potentially untrusted carriers; and (II) to secure the transfer of ownership at each stage of the chain. For the former, grouping proofs in which the tags of the scanned goods generate a proof of “simulatenous” presence can be employed, while for the latter, ownership transfer protocols (OTP) are used. This paper describes enhanced security solutions for both challenges. We first extend earlier work on grouping proofs and group codes to capture resilient group scanning with untrusted readers; then, we describe a modified version of a recently published OTP based on channels with positive secrecy capacity adapted to be implemented on common RFID systems in the supply chain. The proposed solutions take into account the limitations of low cost tags employed in the supply chain, which are only required to generate pseudorandom numbers and compute one-way hash functions.

## 1. Introduction

The National Strategy for Global Supply Chain Security [1] identifies two goals for securing supply chains: (1) promote efficient and secure services, and (2) foster resilience. In particular, the infrastructure should be modernized with security mechanisms integrated into supply chain operations to mitigate vulnerabilities.

**Figure 1:**
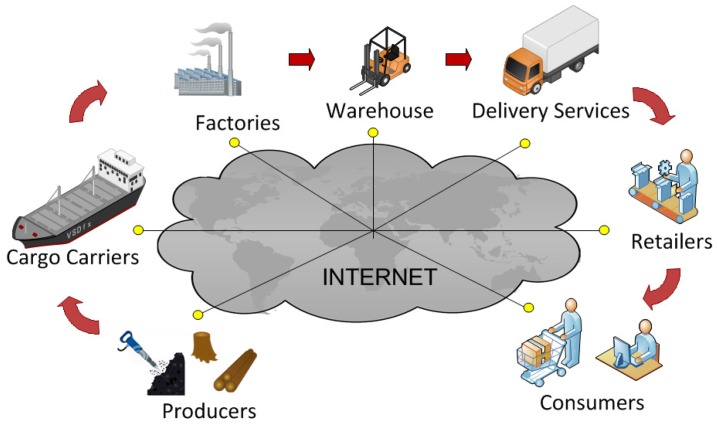
Real-time awareness in the supply chain: flow information is shared at any point of the distribution chain.

Radio Frequency Identification (RFID) is a widely deployed technology for supply chain management, inventory, retail operations and more generally automatic identification. A typical RFID deployment has three main components: tags or transponders, which are electronic data storage devices attached to (or embedded in) objects to be identified; readers or interrogators, that manage tag population, read data from and write data to tags; and a back-end server (or verifier in security applications), which is a trusted entity that exchanges tag information with the readers and processes data according to specific task applications. When combined with the Internet (see Figure 1), RFID technology enables real-time product flow visibility, with information shared at any point in the distribution chain. For example, when a product runs low at the distribution center (detected, for instance, by “smart” shelves with RFID readers), the supplier is automatically alerted to ship more products. Real-time information also allows for accurate ordering. There is no need to keep products piled up in warehouses. Logistics software can be used to trace trucks with GPS (Global Positioning System) locators, while trucks monitor their content with RFID readers. Thus, RFID technology helps to address the three main concerns for efficient supply chain management: (a) inventory inaccuracy, (b) the bullwhip effect (increasing swings in inventory in response to shifts in customer demand along the supply chain), and (c) inventory replenishment rules/policies. The ultimate goal of supply chain management is to optimize the supply chain operation: to deliver goods to the end-customers on time at the lowest cost, while realizing the best profit for the involved agents. This also implies addressing security concerns—in particular, guaranteeing the privacy, integrity and availability of applications, bearing in mind the limited computational capabilities of the employed tags. For the supply chain, low-cost passive UHF (Ultra High Frequency) tags, which operate in the far field with backscatter communication [2], are commonly used. These are computationally constrained and cannot carry out complex cryptographic operations. Readers and verifiers/servers, by contrast, are able to perform complex cryptographic operations.

**Figure 2:**
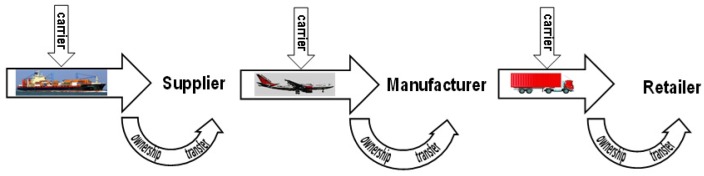
Segments of the supply chain: shipping and ownership transfer.

As goods move along the supply chain from the suppliers to the manufacturers, and then on to retailers, until eventually they reach the end-customers, two types of agents can be identified: the owners, who have complete ownership rights of the goods, and the carriers, who have delegated ownership rights. Thus, the supply chain can be seen as a series of multiple segments in which the current owner (or the seller) of goods ships the goods to a new owner (the buyer) via a carrier (see Figure 2). Then, when goods arrive, the seller must transfer ownership of the goods to the buyer. The security mechanisms (protocols) described in this paper target both phases of these segments: shipping and ownership transfer. We shall assume that owners are trusted, although possibly curious (knowledge of supply chain information can be used to gain competitive advantage), but this trust does not extend to other parties of the chain (e.g., the carriers).

RFID-tagged objects are typically shipped via (potentially untrusted) carriers in pallets. In such cases, it is important that the owner can periodically check the integrity of a shipment. Tags are beyond the communication range of the owner so that the common sequential interrogation of the tags cannot be employed here and grouping proofs can be used instead. A grouping proof involves multiple tags generating a proof of simultaneous presence in the range of an RFID reader [3,4] controlled by a potentially untrusted party. While grouping proofs are designed to provide integrity evidence for complete groups that can be verified by the owner, they do not provide any information about incomplete groups. Group codes, which are stored in the tags and work as a forward error correction mechanism, are combined with grouping proofs in this paper to address this issue. Additionally, grouping proofs usually follow a tag-chaining structure, where each tag authenticates the message coming from the previous tag in the chain. This causes availability issues when “alien” tags, not belonging to the pre-defined group, are involved in the protocols, and privacy concerns, as the total number of tags and the reply order are usually leaked. The grouping proof proposed in this paper avoids the use of this chaining structure implementing a two-round protocol with missing tag identification, which prevents the aforementioned problems.

Ownership Transfer Protocols (OTPs) allow the secure transfer of tag ownership from a current owner to a new owner. Three entities are present in an OTP: the tag T whose rights are being transferred, the current (or previous, after the transaction) owner who has the initial control of T, and the new owner who will take control of T when the protocol is completed. OTPs must incorporate security requirements that protect the privacy of the parties. More specifically, apart from providing anonymity and untraceability against external adversaries, ownership transfer protocols should (I) guarantee the privacy of the previous owners (*forward secrecy*) so that subsequent owners (or an adversary) cannot link a specific tagged object with previous communications, even if the current private information stored on the tag is revealed (e.g., by physical attacks), and (II) guarantee the privacy of the new and future owners (*backward secrecy*) so that, once ownership is transferred, previous owners cannot access, or trace, the tagged product. The latter, when using symmetric cryptography, is particularly challenging [5,6] and most proposed solutions only solve it partially either by:

1. Employing a Trusted Third Party (TTP) to break the trust link between the tag and its current owner (e.g., [7,8]), or by,
2. Assuming an Isolated Environment (IsE) (e.g., [9,10]), without any adversarial interference.

In the first approach, although the use of TTPs could be envisaged for this kind of infrastructure (e.g., acting as certificate authorities), most of the OTP protocols proposed in the bibliography for RFID assume symmetric-keys so that TTPs usually share the master key with the tags, becoming the real holders of the tag’s rights, while the actual owners just share session keys generated by these TTPs. This leads to a centralized architecture that may not be appropriate when tags belong to different authorities/companies. The second approach assumes a weak threat model and, as claimed in [6]: if such protection is adequate, then there is no need for security. More recently, a novel approach [11] has shown that the privacy of the new owner can be guaranteed by using channels with positive secrecy capacity. Such channels can be implemented with noisy tags controlled by the new owner that obfuscate the communication channel for the previous owner.

This paper focuses on protecting the two phases of the supply chain segments, and as main contributions we:

(1) Extend the notion of a grouping proof of integrity to a broader class of applications where items may be missing. The primary concern of the owner of a shipped pallet is to establish its integrity; however, if some tagged items are missing, then the owner wants a list of the missing items and proof that nothing else is missing (resiliency). Thus, based on the work published in [12], we present a two-round anonymous RFID scanning proof that supports tag privacy such that: (a) the verifier (owner) can authorize an untrusted reader (carrier) to scan a group of tagged items and either generate a proof of integrity, or if some tagged items are missing, identify these and prove that nothing else is missing, (b) the authorization is for one only scanning, (c) tagged items are untraceable while the group is not scanned, and (d) only the verifier (owner) can check the proof: unauthorized inspections or forged proofs will not be accepted.
(2) Extend the implementation of positive secrecy capacity channels for provably secure OTP in [11] by using time-slot modulation, similar to the random-slotted medium access control protocol, to make it possible to implement them without requiring multi-level but binary detection.

These security mechanisms proposed for the segments of the supply chain take into account the particular characteristics of RFID systems such as the vulnerability of the radio channel, the constrained power of devices, the low-cost and limited functionality of tags and the request-response operation mode. In particular, tags are only required to generate pseudorandom numbers and compute one-way hash functions.

The rest of this paper is organized as follows. In Section 2, we provide the background for RFID grouping proofs, group codes and OTPs. Section 3 focuses on the shipment link, discussing grouping proof deployments and capabilities, erasure codes, the threat model and presents, along with our design criteria, a two-round anonymous grouping proof of integrity with missing tag identification. Section 4 addresses security concerns during ownership transfer. We describe a provably secure OTP that uses noisy tags to achieve privacy, and introduce a novel way to implement positive secrecy capacity channels adapted for RFID deployments in the supply chain. Section 5 analyzes this implementation, proving that perfect secrecy is achievable, and providing optimal values for real implementations. Finally, in Section 6, we conclude by summarizing the main results.

## 2. Background

### 2.1. Brief Review of Grouping-Proofs

In 2004, Ari Juels defined a new RFID application called a yoking-proof that generates evidence of simultaneous presence of two tags in the range of an RFID reader. This was extended to grouping proofs for multiple tags—see e.g., [13]. Burmester et al. presented in [14], a protocol that achieved anonymity by using randomized pseudonyms for the group identifer, and forward-security by updating the secret keys and the group keys after each session. Huang and Ku [15] presented a grouping-proof for passive low-cost tags that uses a pseudo-random number generator to authenticate flows and a cyclic redundancy code to randomize strings. The protocol has several weaknesses, some of which were addressed by Chien et al. [16] who, in turn, proposed a new grouping-proof. More recently, Liu et al. [3] proposed a grouping-proof for distributed RFID applications with trusted readers. This proof is vulnerable to de-synchronization and privacy leaks [17]. Peris-Lopez et al. [18] proposed guidelines for securing grouping proofs as well as a yoking-proof protocol (for two tags). Most of these protocols propose tag-chaining structures where each tag authenticates the message coming from the previous tag in the chain. This, however, as mentioned, causes privacy problems, as the total number of tags and reply order are leaked, and availability issues when tags that do not belong to the group participate.

**Figure 3:**
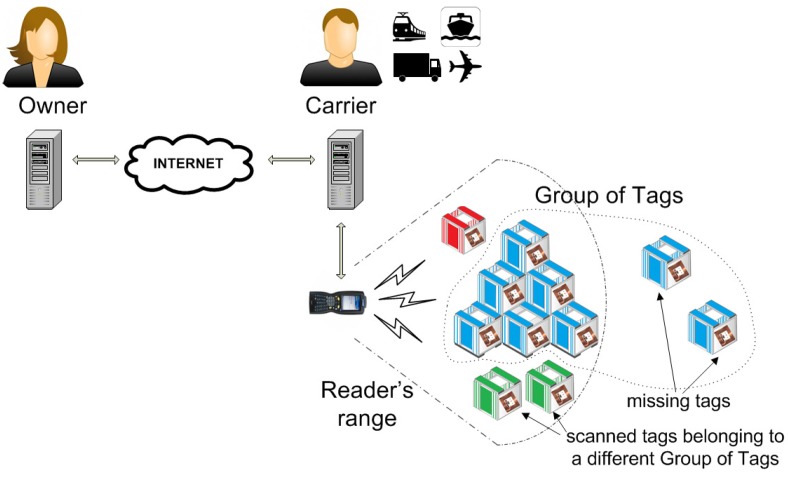
An untrusted carrier can compile a scanning proof of integrity for the tagged goods of a pallet that the owner can verify, and identify any missing tagged goods of the pallet (or, that are beyond the reader’s range).

For the shipment link of the supply chain, the typical deployment of an RFID grouping proof involves: a pallet *P* containing a collection of tagged goods, the owner *Own* of *P* who knows the private information stored by the tags, and a reader that is controlled by the carrier whose services are contracted by the owner and has physical possession of *P*—see Figure 3 for an illustration. In this scenario, if the carrier is trusted, then the owner can entrust the carrier with sensitive information so that the carrier can act as the owner by proxy. The integrity can then be checked by authenticating the different tagged products sequentially. This option is also possible if the owner enjoys full connectivity with the carrier so that the owner can communicate with the tags in real time while the carrier just relays the messages, acting as a communication enabler. The problems arise when the carrier is not trusted and: (a) the owner is only willing to give the carrier information that is strictly needed to ensure the efficient monitoring of the transported goods, and (b) the owner and/or carrier do not enjoy full connectivity. In these cases, grouping proofs are employed. A grouping proof involves a collection of tagged objects generating a proof of simultaneous presence. With low-cost RFID tags, symmetric key cryptography is usually employed so that this proof must be checked by a party that shares private information with the scanned tags, namely *Own*.

### 2.2. Brief Review of Group Codes

**Figure 4:**
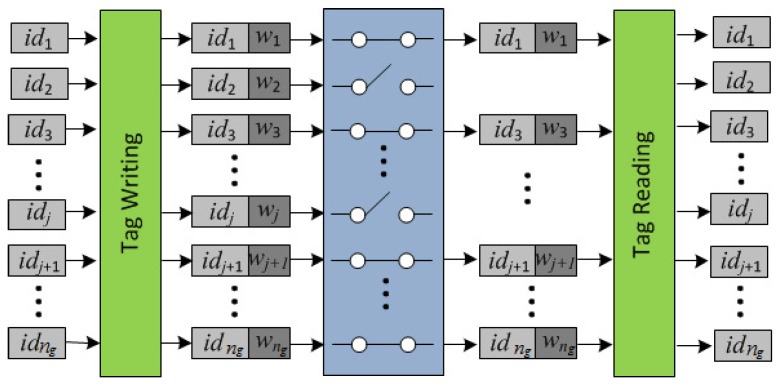
The write-transmit-read process with forward error correction in the supply chain. The loss of tags is modelled using an erasure channel.

While grouping proofs provide integrity evidence for complete groups of tags, they do not address incomplete groups. In particular, they do not provide any information about missing tags. Sato et al. [19] proposed a group code that makes it possible to find the identifiers of missing tags without requiring a packaging list or an external database. More specifically, the missing tags are identified by storing redundant information $w_{i}$ in the memory of each tag. Figure 4 illustrates the write-transmit-read process with forward error correction for supply chain applications. The possible loss of tags is modelled by using an erasure channel. An erasure channel is a memoryless channel that, on inputting a symbol *x*, outputs symbol *x* or no symbol at all. Note that the loss of a tag implies not only the loss of its identifying information $id_{i}$ (when systematic codes are used) but also the loss of the redundancy information $w_{i}$.

Sato et al. [20] use Gallager low-density parity check (LDPC) codes for forward error correction [21]. However, the randomised nature of LDPC codes makes it difficult to get specific decoding guarantees. To address this, Su et al. [22] use strongly selective families (SSF). Su and Tonguz [23] propose a variant that uses the Chinese remainder theorem to construct non-regular generating matrices. Another modification proposed by Su [24] uses resolvable transversal designs to generate parity-check matrices and group splitting to improve performance. Mabrouk and Couderc [25] propose a group code that is based on Reed–Solomon (RS) codes. However, the size of the blocks and the partitioning of the redundancy is not optimal. Burmester and Munilla [26,27] analyze the memory-erasure tradeoff of these group codes and consider optimized approximations for practical settings. They conclude that optimized RS codes are the most efficient from a memory point of view, but impose a higher computational burden on the verifier (reader), particularly when the total number of tagged goods is large. By contrast, LDPC codes are more efficient from a computational point of view, but require considerable more memory that makes them impractical for most RFID applications. Thus, in this paper, we shall assume Reed–Solomon codes to encode tag identifiers.

### 2.3. Brief Review of Ownership Transfer Protocols

Ownership Transfer Protocols (OTP) are intended to transfer of ownership rights of a tag T from a seller or current owner ${\mathrm{Own}}_{c}$ to a buyer or new owner ${\mathrm{Own}}_{n}$. The ownership of a tag usually implies the knowledge of keys that allow to identify and/or access the tag. Before the execution of the OTP, the current owner is the only one that can identify and trace the tag, while after its execution, to guarantee forward secrecy, the tag T can only be identified and traced by the new owner.

The first OTPs for RFID were presented in 2005 by Saito et al. [28] and Molnar et al. [29]. The former describes two proposals: one with TTP, and another without TTP whose security relies on the short range of the backward channel, assuming that it is difficult for adversaries to eavesdrop on this channel. The latter proposes an OTP that, by using a tree structure to manage tag keys, uses a distributed TTP. Ref. [30] analyzes some vulnerabilities of this scheme and a modification that replaces the TTP with distributed local devices is presented by Soppera and Burbridge [31]. Hash values to protect messages and a keyed encryption function combined with a sort of TTP were used by Osaka et al. [32]. This scheme was later modified by Chen et al. [33] and Japinnen and Hamalainen [34] to prevent Denial of Service (DoS) attacks, and by Yoon and Yoo [35] to assume an IsE where the owners can change the tag’s key (some vulnerabilities are described in [36]). RFIDdot, proposed by Dimitriou [37], is an ownership transfer scheme based on random nonces and a keyed encryption function that assumes a private environment where key updates are carried out. An IsE is also assumed by Song and Mitchell [38,39], but they use keyed hash functions and one-time tag identifiers with hash-chains. Ref. [40] defines extended capabilities such as: Tag Assurance, Undeniable Ownership Transfer, Current Ownership Proof, Ownership Delegation, and Authorized Recovery. Ref. [6] proposes two new schemes based on a TTP and IsE, respectively, for ownership transfer of single tags. A version for multiple tags has also been published [41]. More recently, several OTPs that comply with the EPCGen2 standard [42] have been published. These also assume TTPs or IsEs and rely on XOR operations, Cyclic Redundancy Codes (CRC16) and/or Pseudo Random Number Generators as security primitives (e.g., [8,43,44,45]). Some security issues of such proposals are analyzed in [46].

We note that, to guarantee forward secrecy, most of the ownership transfer protocols proposed in the literature rely either on the use of TTPs, or on the assumption of an IsE. Symmetric-key based OTPs that use TTPs often have a centralized management structure that may not be compatible with the distributed management of RFID systems. For example, the RFID parties (the owners) with possibly conflicting interests must trust the TTP that manages their tags. On the other hand, the assumption of IsEs where no adversary can interfere, presumes a weak threat model; Ref. [6] claims that if such an environment were available, then no other security protection would be needed. Moreover, most of the proposed protocols cannot be implemented when the seller and the buyer of shipped tagged goods are in different locations. Recently, a provable secure OTP that addresses these issues has been proposed [11]: this employs a channel with positive secrecy capacity to guarantee the privacy of the new owner, without requiring TTPs or IsEs, and a communication model in which the current owner and the new owner can be in different locations. This paper proposes a modification of this protocol adapted to the ordinary RFID readers used in the supply chain that is based on a binary level instead of multi level detection.

## 3. The Shipment Link

The combined functionality of a grouping proof of integrity with the automatic identification of missing items adds resilience by detecting shrinkages. In this section, we shall describe a pallet scanning proof, defined as a grouping proof with missing tag identification based on work in [12]. In particular, we present an enhanced two-round grouping proof for which the identifiers of the tags are extended to include redundancy, that makes it possible to identify missing tagged objects and prevent availability (when “alien” tags participate) and privacy issues (reply order leakage). Additionally:

1. The owner of the pallet *P* (e.g., the supplier, manufacturer, retailer, etc.) can authorize an untrusted carrier to inspect *P* for integrity and identify any missing goods.
2. The authorization is for a certain number of inspections (or limited time) defined by a counter $T_{s}$, and the contents of *P* are untraceable after the authorization expires. In particular, the carrier does not share any private keys with the tags and cannot access or even trace the tags beyond the lifetime of the counter $T_{s}$.
3. The carrier can generate a grouping proof of integrity for the pallet *P* that (only) the owner can verify if no goods are missing; if some goods are missing, then the carrier can (a) identify the missing goods without requiring a packing list (or an external database) and (b) generate a scanning proof of presence for the remaining goods.
4. The grouping proof is generated only if the tags of the group were scanned simultaneously (during the same session defined by the activation time of the tags) within a time window defined by $T_{s}$.

For the design of the scanning proofs, the following assumptions are made:

a *The tags of a pallet are not compromised*. This does not mean that tags cannot be compromised; but if they are, then the corroborating evidence generated for a scanning proof is compromised.
b *Simultaneity*. This is defined in terms of counters or timestamps provided by the owner.
c *Batch connectivity*. The owner does not enjoy permanent connectivity with the carrier and is restricted to: (a) broadcasting a challenge that is valid for a (short) time span and, (b) checking responses from tags that are compiled and sent from time to time by untrusted readers.
d *Balanced loading*. The tags of a pallet have similar hardware capabilities and the computation load per tag is balanced.
e *Messages must include destination information (possibly private) to allow for unicast/multicast communication.* This is sometimes neglected by designers, but it is particularly important for checking anonymity: each message must contain information that allows tags to decide if they are the intended recipient.

### 3.1. Extended Identifiers with Redundancy

We shall use a Reed–Solomon RS(n,k) code over $F_{2^{m}}$, 2≤m≤16 (in compliance with RFC 6865 [47]) to encode the identifiers $\left(id_{1},\ldots ,id_{n{\;}_{g}}\right)$ of a collection of $n_{g}$ RFID tags, so as to recover up to $s_{t}=\;\left(n\;-\;k\right)/\left(n/n_{g}\right)$ missing identifiers $id_{i}$. For this purpose, we rearrange the source data $x=id_{1}\;\;∥\;\cdots \;∥\;\;id_{n{\;}_{g}}$, which is an $n_{g}\ell$-bit string, where *ℓ* is the binary length of the identifiers $id_{i}$, into *k* blocks of size *m*: $x=(x_{i},\ldots ,x_{k})$ (so $x_{i}\in F_{2^{m}}$), and then encode *x* to get an *n* block codeword $y=(y_{1},\ldots ,y_{n})$ (depending on the implementation, some blocks $x_{i}$ can be padded with zeros if necessary). The codeword *y* is then partitioned into $n_{g}$ pieces of size $n/n_{g}$ blocks, denoted ${\mathrm{ID}}_{i}$, which are stored (written) to the memory of each $tag_{i}$. The ${\mathrm{ID}}_{i}$ contain the identifying information $id_{i}$ as well as redundancy $w_{i}$ (the systematic property of linear error-correcting codes allows the separation of source and redundancy information) needed to recover erased blocks. This code will recover up to s=n-k blocks $y_{i}$, which corresponds to $s_{t}=floor\left(\left(n-k\right)/\left(n/n_{g}\right)\right)$ missing identifiers $id_{i}$. To identify the missing tagged products, the data collected (read) from the tags is used to generate a codeword $y^{*}$ with erasures. To decode $y^{*}$, we need to order the scanned identifiers ${\mathrm{ID}}_{i}$ correctly so that *y* and $y^{*}$ agree on all non-erased positions. For this purpose, control information is used: the information stored in each tag is extended to include a few bits that define its order *i* when it was encoded in the codeword *y*.

### 3.2. Scanning Proof Description

**Figure 5:**
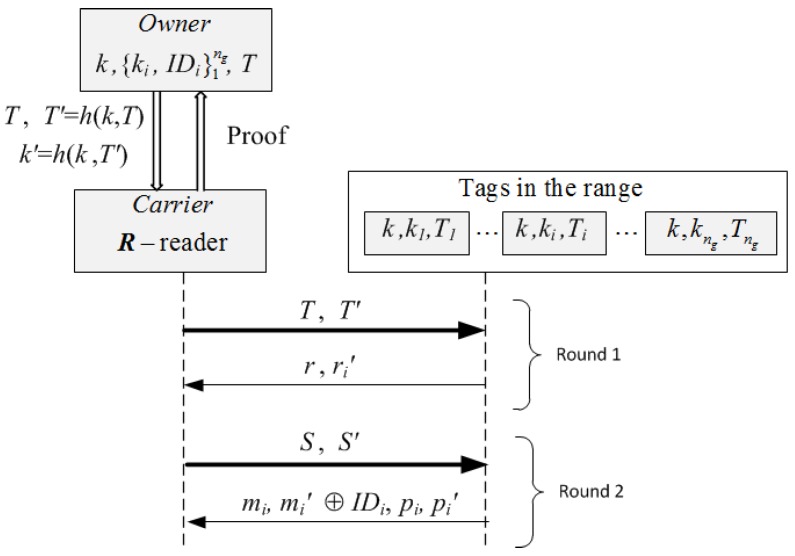
The two rounds of the anonymous scanning proof with missing tag identification.

Let $P=\{tag_{1},\ldots ,tag_{n_{g}}\}$ be a collection of tags attached to the goods of pallet and *h* denote a cryptographic hash function. The owner of each collection *P* stores the tuple: $\left(T,k,{\left\{\left(k_{i},{\mathrm{ID}}_{i}\right)\right\}}_{i\in [1:n_{g}]}\right)$, where *T* is a counter value, *k* is a key for the collection of tags, $k_{i}$ is the private key of $tag_{i}$, and ${\mathrm{ID}}_{i}$ is the extended identifier of $tag_{i}$ that includes its identifying code $EPC_{i}$ and redundant information $w_{i}$ used to recover missing tags. Each $tag_{i}$ of *P* stores in non-volatile memory: $\left(k,k_{i},{\mathrm{ID}}_{i}\right)$ and a counter $T_{i}$ that is initialized to the same value $T_{0}$ for all tags of *P*. The carrier’s reader *R* initially does not share any information with the tags of *P*, and the process starts when *R* receives from the owner a scanning request $\left(T,T^{\prime },k^{\prime }\right)$, where: *T* is a fresh value of a counter, $T^{\prime }=h\left(k,T\right)$ is a session authenticator, and $k^{\prime }=h\left(k,T^{\prime }\right)$ is the session key. Then, a two rounds protocol takes place in which the reader and the tags generate a scanning proof with missing tag identification (see Figure 5):

**Round** **1.** The reader *R* of the carrier broadcasts to all tags in its range: $\left(T,T^{\prime }\right)$, and sets a timer. Each $tag_{i}$ in the range of *R* computes $k^{\prime }=h\left(k,T^{\prime }\right)$ and checks the correctness of $T^{\prime }$ by verifying that $T^{\prime }=h\left(k,T\right)$ and that the counter value $T>T_{i}$. If any of these fail, $tag_{i}$ returns two random values. Otherwise, it updates its counter to *T*, draws a random number $r_{i}$ and computes its authenticator $r_{i}^{\prime }=h\left(k^{\prime },r_{i}\right)$. Then, it sends $\left(r_{i},r_{i}^{\prime }\right)$ to *R* and sets a timer. The received nonces $r_{i}$ are used by the reader *R* to identify (singulate) tags in this session (session pseudonyms). *R* checks the correctness of every $r_{i}$ by verifying that $r_{i}^{\prime }=h\left(k^{\prime },r_{i}\right)$, and if this holds, *R* stores them in a list L1. On timeout, *R* computes the request $S=h(T,r_{j_{1}},\ldots ,r_{j_{u}})$, where $\left\{j_{1},\ldots ,j_{u}\right\}\subseteq \left\{1,\ldots ,n_{g}\right\}$ are the indices of the tags of pallet *P* that were scanned, and its authenticator $S^{\prime }=h\left(k^{\prime },S\right)$. Thus, the first round incorporates the randomness provided by the owner’s challenge *T* and the randomness $r_{i}$ provided by the interrogated tags. This prevents replay attacks. The participation of “alien" tags does not affect the execution (availability is guaranteed) and information about the total number of tags or reply order is not leaked because tags do not follow any chaining structure. The scanning period is defined by the scanning request *T* of the reader, and simultaneity by the validity period of the nonces $r_{i}$ that is set by the scanned tags.
**Round** **2.** The reader *R* broadcasts the authenticated request $\left(S,S^{\prime }\right)$ to all tags in its range. Each $tag_{i}$ in the range of *R* that has not timed out, checks that $S^{\prime }=h\left(k^{\prime },S\right)$ and if so, it computes: $m_{i}=h\left(k^{\prime },r_{i},{\mathrm{ID}}_{i}\right)$ and its session authenticator $m_{i}^{\prime }=h\left(k^{\prime },m_{i}\right)$, as well as a “proof of presence during the session” $p_{i}=h\left(k_{i},r_{i},S\right)$ (a message authentication code), and its authenticator $p_{i}=h\left(k^{\prime },p_{i}\right)$. Then, it encrypts its identifier ${\mathrm{ID}}_{i}$ with the “one-time-pad” key $m_{i}^{\prime }$ to get $m_{i}^{\prime }⊕{\mathrm{ID}}_{i}$, sends to *R*: ($m_{i}$, $m_{i}^{\prime }⊕{\mathrm{ID}}_{i}$, $p_{i}$, $p_{i}^{\prime }$), and timeouts. The reader *R* computes $m_{i}^{\prime }=h\left(k^{\prime },m_{i}\right)$ and retrieves the identifiers ${\mathrm{ID}}_{i}$. Then, it checks (by exhaustive search) that $m_{i}=h\left(k^{\prime },r_{i},{\mathrm{ID}}_{i}\right)$ for some value $r_{i}$ in the list L1, and that $p_{i}^{\prime }=h\left(k^{\prime },p_{i}\right)$. If these are correct, *R* stores the identifiers ${\mathrm{ID}}_{i}$ in a list L2. On timeout, *R* checks that |L1|=|L2| (that all tags singulated in Round 1 responded in Round 2), and if so, compiles the proof $W=(T,{\mathrm{ID}}_{j_{1}},\ldots ,{\mathrm{ID}}_{j_{u}},r_{j_{1}},\ldots ,r_{j_{u}},h\left(p_{j_{1}},\ldots ,p_{j_{u}}\right))$ as evidence that the tags were scanned. Otherwise, *R* aborts the protocol. Then, using the control information, *R* checks that the cardinality of the group coincides with |L2|. If not, *R* finds the missing $EPC_{i}$s by using the redundant information stored in the retrieved identifiers ${\mathrm{ID}}_{j}$, provided that this is within the correction capabilities of the implemented forward error correction mechanism (i.e., the number of missing tags $\left(n_{g}-u\right)$ is no more than $s_{t}=\left(n-k\right)/\left(n/n_{g}\right)$). If there are no missing tags, then *W* becomes a grouping proof of integrity for pallet *P* that the reader *R* sends to the owner *Own*. Otherwise, *R* retrieves the list of identifiers $EPC_{i}$, $i\in \left\{1,\ldots ,n_{g}\right\}\setminus \left\{j_{1},\ldots ,j_{u}\right\}$, of the missing goods, and sends *Own* the scanning proof $W^{*}=\left(T,{\mathrm{ID}}_{j_{1}},\ldots ,{\mathrm{ID}}_{j_{u}},r_{j_{1}},\ldots ,r_{j_{u}},h\left(p_{j_{1}},\ldots ,p_{j_{u}}\right)\right)$ of presence for the remaining goods.

To validate the scanning proof, *Own* first uses the value *T* to retrieve the tuple $\left(k,{\left\{k_{i},{\mathrm{ID}}_{i}\right\}}_{i\in [1:n_{g}]}\right)$. Then, *Own* computes $S=h(T,r_{j_{1}},\ldots ,r_{j_{u}})$ using the values $r_{j_{i}}$ given by the carrier and the corresponding $p_{j_{i}}=h\left(k_{j_{i}},r_{j_{i}},S\right)$. Finally, *Own* checks that the value of $h(p_{j_{1}},\ldots ,p_{j_{u}})$ is correct.

### 3.3. Security Discussion

1. *Traceability attacks (privacy)*. Unlinkability is related to the capability of linking interrogations after physical tracking is temporarily interrupted. Different formal models can be found in the literature (e.g., [48,49,50]). Intuitively, a protocol guarantees unlinkability, if no adversary can decide with advantage better than negligible whether two transmitted messages from different protocol executions are linked to the same tag T. In the scanning proof, $tag_{i}$ is untraceable because, in every session, it updates its counter $T_{i}$ and will draw a fresh (pseudo) random number $r_{i}$ after responding to the reader’s challenge *T*. Consequently, the responses of the same tag in different interrogations look random to an observer and cannot be linked. Tags do not follow a sequence to reply so that information about the order of a tag cannot be leaked.
2. *Replay Attacks*. The use of the counter *T* prevents replay attacks: if an adversarial reader re-uses *T*, the tags that received it earlier will have updated their counter and not respond.
3. *Impersonation attacks*. Impersonation attacks on tagged goods are prevented by using private keys $k_{i}$. Impersonation attacks on a reader will not yield a valid proof since the owner will only accept proofs from authorized readers that have been given $\left(T,T^{\prime },k^{\prime }\right)$ .
4. *Forged proofs.* The values $p_{i}=h\left(k_{i},r_{i},S\right)$ can only be generated by someone who knows $k_{i}$; i.e., $tag_{i}$ and the owner. Values $p_{i}$ from different sessions cannot be used to compile a proof since they involve the session nonces $r_{i}$ of interrogated tags and the challenge of the reader *R* ($=h(T,r_{j_{1}},\ldots ,r_{j_{u}})$) that depends on the counter *T* which specifies the validity time window. Note that all tags set timers in Round 1 of the protocol and will not respond after timeout.
5. *De-synchronization attacks (DoS attacks)*. The adversary cannot compute a valid pair of values $\left(T,T^{\prime }\right)$ because this requires knowledge of the key *k*. On the other hand, if a protocol execution completes successfully, then all tags will share the same counter value. No tag will accept a previously used *T*. However, tags will accept future values of *T*, not necessarily the next value, so that even if they do not share the same counter value (e.g., because of an interrupted interrogation), there are no synchronization concerns.

Two possible bottlenecks for tag populations can be identified: (I) in time terms, the exhaustive search in Round 2 to check that $m_{i}=h\left(k^{\prime },r_{i},ID_{i}\right)$ for values $r_{i}$ in list L1, and (II) in memory overhead, the extra bits stored in the tags as required by grouping codes. Only tags that know $k^{\prime }$ are included in L1 so that in normal conditions, for a low number of missing tags, these factors should not be a problem, even for large groups. However, when the rate of missing tags increases, the second factor could limit tag population [26]. In fact, for groups of about 100 tags, 12 and 144 extra bits are required for missing tag rates of 10% and 60%, respectively. Therefore, the tag population is expected to be limited not by the anonymization, but by the use of grouping codes and the expected missing tags rate. Note, however, that very large groups are not usually assumed for grouping proofs since, unlike the two-round protocol presented here, most of the previously published grouping proofs follow a sequential tag-chaining structure where each tag in the group authenticates a message coming from the previous tag in the chain.

## 4. Ownership Transfer Link

The provable secure OTP described in [11] captures spatiotemporal requirement so that it is appropriate for applications such as the supply chain where the seller and buyer are in different locations. The protocol has two phases. Initially, the tag T whose ownership is going to be transferred shares a private key $k_{0}$ with ${\mathrm{Own}}_{c}$. In the first phase, a new fresh key $k_{1}$ is agreed between ${\mathrm{Own}}_{c}$, T and ${\mathrm{Own}}_{n}$. For this purpose, ${\mathrm{Own}}_{c}$ first exchanges privately the key $k_{1}$ with the tag. Then, ${\mathrm{Own}}_{c}$ sends privately to ${\mathrm{Own}}_{n}$ the key $k_{1}$. Finally, T and ${\mathrm{Own}}_{n}$ confirm mutually knowledge of $k_{1}$ and T updates its private key $k_{0}$ to $k_{1}$. This completes the first phase (see [11] for more details). Forward secrecy is guaranteed because $k_{1}$ does not provide ${\mathrm{Own}}_{n}$ with any information about $k_{0}$. However, the key $k_{1}$ shared by ${\mathrm{Own}}_{n}$ and T is also known to ${\mathrm{Own}}_{c}$, who can use it to keep tracking the tag, violating backward privacy. The second phase of the protocol addresses this issue.

The second phase involves a Key Update Protocol (KUP) that exploits signal features at the physical layer to create a channel with positive secrecy capacity. In particular, “noisy tags” controlled by the new owner are used to obfuscate the previous owner’s channel so that the new owner and the tag can exchange a new secret $k_{2}$ without the previous owner being able to access it [51]. Before going into technical details, to understand the idea behind this protocol, let us consider a scenario that involves a crowd of people all calling out “yes” or “no” (the noisy tags) to obfuscate the decision (“yes” or “no”) of an oracle (the tag) from an eavesdropper (the previous owner). The eavesdropper will only know with absolute certainty the decision of the oracle if all calls (crowd + oracle) were “yes” or “no”. In the other cases, the eavesdropper only knows the decision with varying degrees of certainty depending on the tally of the calls (crowd + oracle). However, anyone who knows the tally of the crowd (the new owner) can disambiguate the oracle’s decision by subtracting the tallies. In this way, a person can send one bit (“yes” or “no”) of information privately to a listener who knows the bit values of the calls made by the crowd, while the eavesdropper only gets the bit with a certain probability. The difference between the information that the listener and eavesdropper get is called the *secrecy capacity*. The noisy tags create a communication channel with positive secrecy capacity that can be used by the new owner and the tag to exchange information privately. Thus, in the KUP proposed in [11], tag T and the noisy tags $T^{*}$ are queried by the new owner with a random number *r*, and all respond at the same time with *S* and $S_{i}^{*}$, respectively. The new owner and the eavesdropper (the previous owner) receive the sum of the strings *S* and $S_{i}^{*}$, but only the new owner who knows $S_{i}^{*}$ is able to extract *S*. The new owner and the tag then use this value to compute a new key $k_{2}$ (the full proof is in [11]).

**Figure 6:**
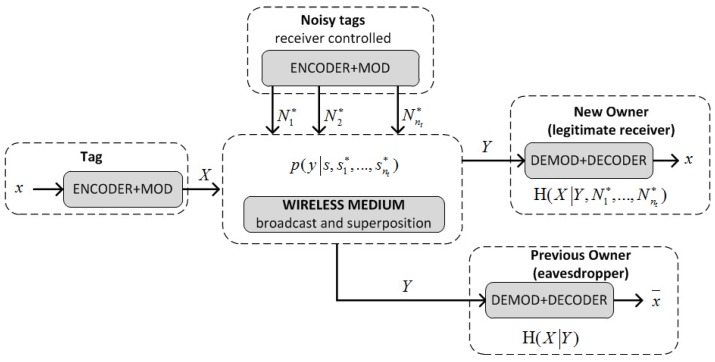
A model for the wiretap channel with noisy tags.

The physical addition of the signals corresponding to *S* and $S_{i}^{*}$ in the channel is the basis of the wiretap channel (see Figure 6). *X* and $N_{i}^{*}$ are random input variables taking values *s*, $s_{i}^{*}$ in the input alphabet X. *Y* is an output random variable taking value *y* in the output alphabet Y, and $p(y|s,s_{1}^{*},\ldots ,s_{n_{t}}^{*})$ is the transition probability of the channel. Tag T transmits the message X=x to the new owner (the intended receiver) with the help of $n_{t}$ noisy tags, in the presence of the current (previous) owner ${\mathrm{Own}}_{c}$, who acts as a passive eavesdropper. The wiretap channel is a stochastic encoder of *X* with output *Y*. *Y* is input to the maximum a posteriori probability (MAP) estimators of ${\mathrm{Own}}_{n}$ and ${\mathrm{Own}}_{c}$, but while ${\mathrm{Own}}_{c}$ only knows the value of *Y*, ${\mathrm{Own}}_{n}$ also knows the values of the inputs $N_{1}^{*},\ldots ,N_{n_{t}}^{*}$. The wireless medium is assumed noiseless, so that the estimate X=x of ${\mathrm{Own}}_{n}$ is correct while the estimate $X=\bar{x}$ of ${\mathrm{Own}}_{c}$ is degraded by the stochastic encoder. The degradation is quantified by the conditional entropy H(X|Y):

(1) $$H\left(X|Y\right)\;=\;\sum_{j=0}^{|X|-1}\sum_{k=0}^{|Y|-1}-p\left(x_{j},y_{k}\right)\cdot \log_{2}p\left(x_{j}|y_{k}\right)\;\;.$$

The capacity of the eavesdropper channel (${\mathrm{Own}}_{c}$’s) is defined as $C_{eav}=H\left(X\right)-H\left(X|Y\right)$. The secrecy capacity for the wiretap model is $C_{s}=C_{\mathrm{main}}-C_{eav}$, where $C_{\mathrm{main}}$ is the capacity of the main channel (${\mathrm{Own}}_{n}$s). In the noiseless case, we have $C_{\mathrm{main}}=H\left(X\right)$, and therefore the secrecy capacity coincides with the conditional entropy of the eavesdropper $C_{s}=H\left(X|Y\right)$. In particular, if the source is binary and equiprobable (H(X)=1) and the length of *S* and $S_{i}^{*}$ is $n/C_{s}$ bits, then ${\mathrm{Own}}_{c}$ knows $C_{eav}\cdot n/C_{s}=\left(1-C_{s}\right)\cdot n/C_{s}$ bits of *S*, while the remaining *n* bits are unknown. These bits are used by T and ${\mathrm{Own}}_{n}$ to compute the new key $k_{2}$ so that, once the KUP is completed, ${\mathrm{Own}}_{c}$ has no control over the tag T and cannot trace it.

**Figure 7:**
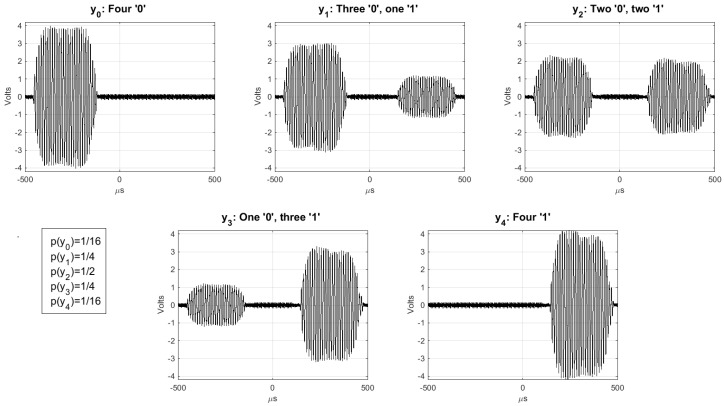
Examples of the output alphabet $Y=\{y_{0},y_{1},y_{2},y_{3},y_{4}\}$ of the modulation described in [11] with $n_{t}=3$.

In [11], the performance for different values of $n_{t}$ is analyzed, proving that $\lim_{n_{t}\to \infty }H\left(X|Y^{n_{t}}\right)=H\left(X\right)$ (perfect secrecy), and showing that $n_{t}=3$, with $C_{s}=0.78$, is a good compromise for ease of implementation and performances. Figure 7 shows the outputs of Y for the KUP described in [11] with $n_{t}=3$. For this implementation, the tags are assumed to respond quasi-simultaneously (or without distinguishable delays) and the readers to demodulate output symbols with multiple amplitude levels. This is equivalent, in the example above, to the listener being able to identify the specific number of people that responded simultaneously “yes” and “no”. Such implementations, however, may not be practical for the supply chain where binary RFID readers are preferred. That is, the listener only needs to detect if someone is saying “yes” or “no”, but not the number of people that simultaneously are saying it. In the next section, we shall describe a modified version of this KUP that is adapted for the common binary RFID systems employed in the supply chain. More specifically, this employs a channel with positive secrecy capacity that circumvents the need for tags to respond quasi-simultaneously, and for readers to distinguish amplitude levels.

## 5. A KUP That Uses a Positive Secrecy Capacity Channel Adapted for the Supply Chain

### 5.1. A Positive Secrecy Capacity Channel Based on Modified Random-Slotted Modulation

The EPCGen2 standard [42] specifies the Random-Slotted Collision Arbitration algorithm, which is a modified version of the Frame Slotted Aloha protocol, for random access in RFID systems. In this protocol, the reader sends to all tags in its range a query *Q* and tags pick a random value in the range $\left(0,\ldots ,2^{Q}-1\right)$ to respond in that slot. As Miller coding is used, when two or more tags chose the same slot to transmit different values, a collision is detected. Otherwise, the reader detects the transmitted information. Based on this scheme, we shall now describe how to implement a channel with positive secrecy capacity that does not require multilevel detection.

**Figure 8:**
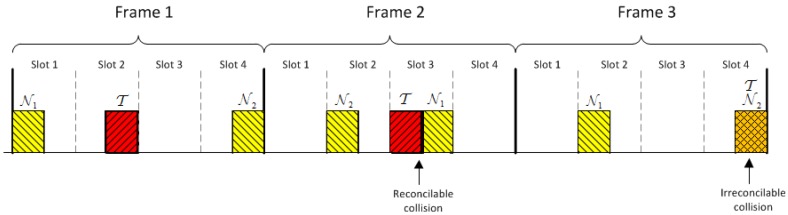
Examples of output symbols with the modified random-slotted modulation mechanism.

In the proposed system, bits are transmitted in frames of *f* slots. To transmit a bit, a tag picks one of these slots and uses it to transmit the bit. Figure 8 illustrates an example with three frames when f=4, $n_{t}=2$ and Manchester coding is used. For the sake of simplicity, we have preferred here to use Manchester coding rather than Miller coding as this depends on the previous bit, but both coding allow detecting collisions. In the example, T transmits the bit string ‘101’ using the slots 2, 3 and 4, respectively. Likewise, the noisy tag $N_{1}$ transmits the bit string ‘010’ using the slots 1, 3 and 2, and the noisy tag $N_{2}$ transmits the ‘101’ using the slots 4, 2 and 4. By knowing the values transmitted by the noisy tags, the reader can disambiguate the values transmitted by T in the first and the second frames, but it cannot determine it in the third frame because it cannot determine if T sent 0 in the slot 2 or 1 in the slot 4. Thus, we have, in the first frame, three singletons $s_{s}=3$, in the second frame one singleton $s_{s}=1$ and a reconcilable collision $s_{c}=1$, and in the third frame a singleton and an irreconcilable collision, but the readers wrongly detects them as two singletons $s_{s}=2$. It is true that if all tags send the same value (e.g., $N_{1}$ had also sent another 1 in the slot 3), then the reader can determine the value sent by T, but this does not contribute to the secrecy capacity of the channel as the eavesdropper would also know with certainty the value sent by T. As a result, frames with $s_{s}+2s_{c}<n_{t}+1$ are discarded. When these output symbols are removed, the original output alphabet Y is reduced to an alphabet $Y^{\prime }$ of size:

(2) $$|Y^{\prime }|=\left(\begin{array}{c} 2f\\ n_{t}+1 \end{array}\right),\mathrm{with}\;\;\;2f\geq n_{t}+1.$$

We next analyze the performance of this channel for two coding schemes. In the first, as in the example above, the order of the slot is not used in the code so that |X|=2; H(X)=1. In the second, by contrast, the order of the slot is used to code the input (Pulse Position Modulation) so that |X|=2f; $H\left(X\right)=\log_{2}2f$. The secrecy capacity for the first coding can be computed as follows (see the Appendix for more details):

(3) $$C_{s1}\;=\;2{\left(\begin{array}{c} 2f\\ 2f-n_{t}-1 \end{array}\right)}^{-1}\;\sum_{k=1}^{|Y^{\prime }|}\frac{W_{k}^{0}}{n_{t}+1}\log_{2}\frac{n_{t}+1}{W_{k}^{0}}\;\;,$$

where $W_{k}^{0}$ is the number of 0’s in the output symbol $y_{k}^{\prime }\in Y^{\prime }$. The secrecy capacity for the second coding is:

(4) $$C_{s2}=\log_{2}\left(n_{t}+1\right).$$

**Figure 9:**
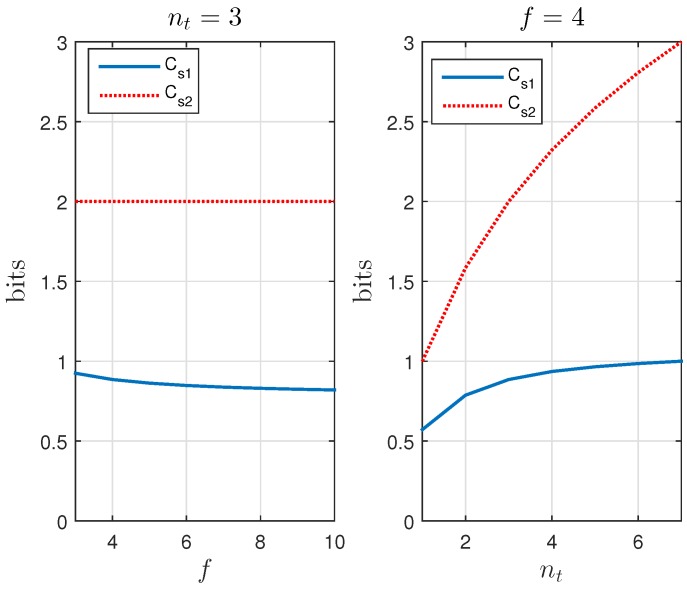
Comparison of the secrecy capacities $C_{s1}$ (|X|=2) and $C_{s2}$ (|X|=2f).

**Figure 10:**
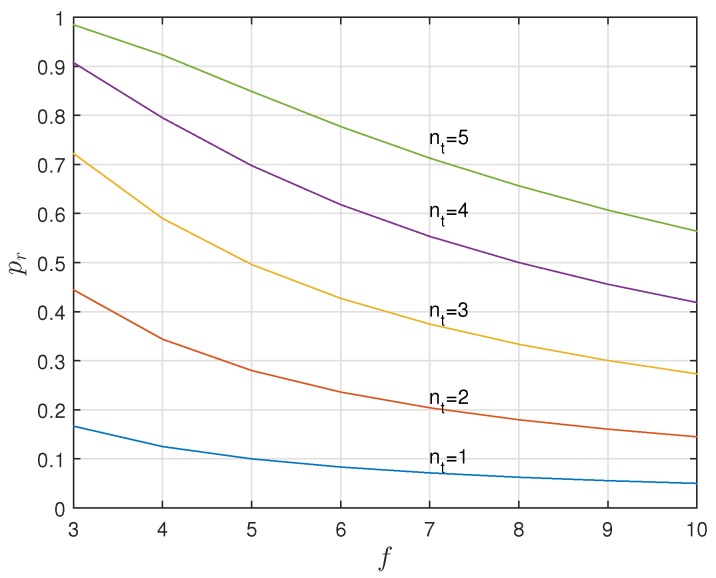
The probability of retransmission $p_{r}$ increases with $n_{t}$ and decreases with *f*.

Figure 9 compares, for different values of $n_{t}$ and *f*, $C_{s1}$, $C_{s2}$. Secrecy capacity increases with the number of noisy tags but not with the number of slots. Perfect secrecy, $C_{s1}=H\left(X\right)$ and $C_{s2}=H\left(X\right)$, is achieved when $n_{t}$ reaches its limit $n_{t}=2f-1$. This may mislead us to infer that reducing *f* improves the efficiency of the system, which is not true because the probability of frame rejection, or frame retransmission, for having irreconcilable collisions does increase when reducing *f*. Thus, perfect secrecy capacity when $n_{t}=2f-1$ involves very high probabilities of retransmission. The probability of retransmission $p_{r}$ is plotted in Figure 10 and can be computed as:

(5) $$p_{r}\;=\;1-\frac{(n_{t}+1)!}{\;{\left(2f\right)}^{n_{t}+1}}\left(\begin{array}{c} 2f\\ 2f-n_{t}-1 \end{array}\right).$$

Frame retransmissions not only increase the communication cost but also the computational cost, since a new value has to be generated (otherwise, the adversary can track tags by checking the repeated values in irreconcilable collisions). Thus, the time to generate and transmit a symbol in a frame slot is:

(6) $$T\;=\;f\cdot t_{f}+t_{c}\cdot log_{2}\left|X\right|\;,$$

where $t_{f}$ is the duration of a frame slot and $t_{c}$ the processing time to generate a new bit. This time *T* is then multiplied by a factor ${\left(1-p_{r}\right)}^{-1}$ to take into account the probability of retransmission:

(7) $$T_{t}\;=\;T\left(1-p_{r}\right)\sum_{i=1}^{\infty }i\cdot p_{r}^{i-1}=\frac{T}{\left(1-p_{r}\right)}\;\;.$$

To understand how these different parameters conjugate, the number of secret bits transmitted per time unit $t_{f}$ can be computed as:

(8) $$C_{f}\;=\;\frac{C_{s}\left(1-p_{r}\right)}{100\;log_{2}\left|X\right|+f+1}\;\;,$$

where $t_{c}\approx 100t_{f}$ has been assumed. Accurate approximations for the latter assumption can be made in each particular case taking into account the particular characteristics of the tag, the quality of the communication link, and the selected operation mode.

**Figure 11:**
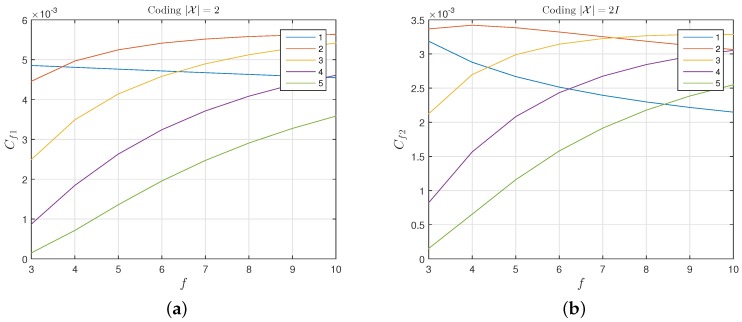
The number of secret bits transmitted per time unit $C_{f}$ for a coding: (**a**) with |X|=2, and (**b**) with |X|=2f.

Figure 11 shows the number of secret bits transmitted per unit time $t_{f}$ when different values of $n_{t}$ and *f* are employed. As a result, the coding with |X|=2 is more efficient than the coding with |X|=2f. This happens because, although, with the latter, more bits are transmitted per frame, the rate $C_{s}/H\left(X\right)$ is lower, and therefore a smaller fraction of the generated bits becomes part of the transmitted secret information. For the code with |X|=2, we identify the set of parameters: $n_{t}=2$, f=8 as offering an optimal compromise between performance and easiness of implementation. For these parameters: $C_{s}=0.73$, $p_{r}=0.18$.

### 5.2. A KUP Based on a Positive Secrecy Capacity Channel with Modified Random-Slotted Modulation

**Figure 12:**
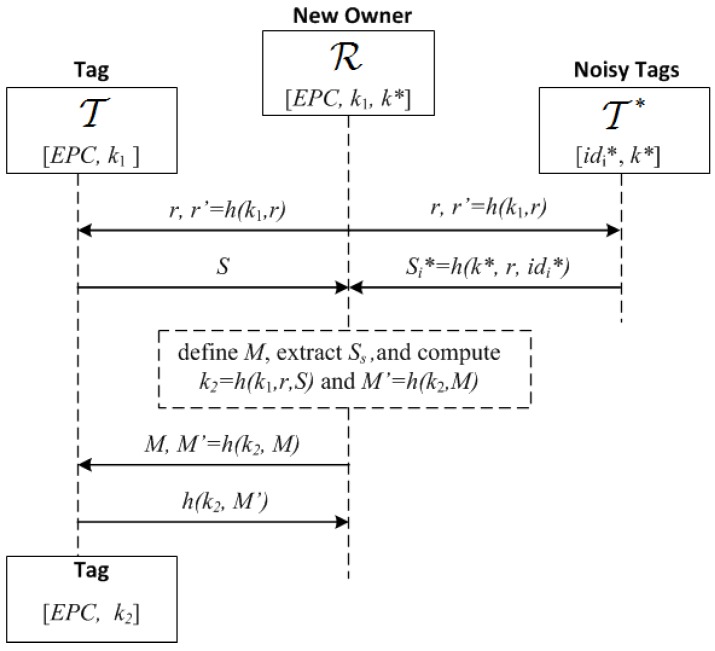
A Key Update Protocol based on a positive secrecy capacity channel with modified random-slotted modulation.

The parties are: the reader R of the new owner, a tag T, and $n_{t}$ noisy tags $T_{i}^{*}$, $i=1,\ldots ,n_{t}$. R shares with T a private key $k_{1}$ and with each $T_{t}^{*}$ a private key $k^{*}$. The goal in this protocol is for T to update privately the key $k_{1}$ with a fresh key $k_{2}$ of length *n* bits while the previous owner, who also knows $k_{1}$, eavesdrops on the communication. The description of the protocol is as follows (see Figure 12):

1. R broadcasts $r,r^{\prime }$, where *r* is a nonce and $r^{\prime }=h\left(k_{1},r\right)$: $R\;\to \;T,\;{\left\{T_{i}^{*}\right\}}_{i=1}^{n_{t}}:\;r,r^{\prime }=h\left(k_{1},r\right).$
2. Upon receiving this, T and $T_{i}^{*}$ check that $r,r^{\prime }$ are correct, and if so, generate a random bit string *S* and the bit strings $S_{i}^{*}=h\left(k^{*},r,id_{i}^{*}\right)$, of length $L=\lceil nF_{g}/\left(C_{s}\left(1-p_{r}\right)\right)\rceil$, where $F_{g}\geq 1$ is a guard factor (e.g., $F_{g}=1.1$). Then T and $T_{i}^{*}$ broadcast these bit strings using a frame for each bit and picking random slots within such frames as described previously (Section 5.1): T and ${\left\{T_{i}^{*}\right\}}_{i=1}^{n_{t}}\;\to \;R:\;S$ and ${\left\{S_{i}^{*}\right\}}_{t=i}^{n_{t}}.$
3. R receives the added signals of *S* and ${\left\{S_{i}^{*}\right\}}_{i=1}^{n_{t}}$. First, R identifies the frames with irreconcilable collisions (by checking that $s_{s}+2s_{c}<n_{t}+1$) and stores their indices in a list *U*. Let $\bar{U}=\left\{1,2,...,L\right\}\setminus U$ be the set of frames without irreconcilable collisions. R generates a bit string $S_{s}$ of length $|\bar{U}|$ with the values of *S* for the frames with indices in $\bar{U}$, and a bit string *M* of length *L*, whose *i*-th bit is 0 if i∈U and 1 if $i\in \bar{U}$. Note that the expected value of $|S_{s}|$: $E[|\bar{U}\left|\right]=L\cdot \left(1-p_{r}\right)=nF_{g}/C_{s}$, is greater than $n/C_{s}$. However if $|S_{s}|<n/C_{s}$, then R generates another random number *r* and repeats the first step, extracting a new $S_{s}$, and concatenating it to the previous one until $|S_{s}|\geq n/C_{s}$. Then, R computes $k_{2}=h\left(k_{1},r,S_{s}\right)$, and sends $M,M^{\prime }=h\left(k_{2},M\right)$:
  $R\;\to \;T:\;M,M^{\prime }=h\left(k_{2},M\right).$
4. T generates $S_{s}$ by taking the bits of *S* where *M* is equal to 1, computes $k_{2}=h\left(k_{1},r,S_{s}\right)$ and checks the correctness of the received $M^{\prime }$. If this is not correct, then T aborts the protocol; otherwise, it computes $h(k_{2},M^{\prime })$ and sends this to R to confirm that the updating was correct:
  $T\to R:h(k_{2},M^{\prime })$.
5. R checks the received message. If correct, the protocol is completed, and the current owner informs the previous one that the process has been completed. Otherwise, R resends the values $M,M^{\prime }$ in Step 3 to checks if T has updated its key. If not, the KUP is repeated.

Security against an eavesdropper comes from the fact that even if the key $k_{1}$ is known, the key $k^{*}$ of the noisy tags is not known, and therefore, the adversary cannot filter out $S_{i}^{*}$ to get $S_{s}$ and compute $k_{2}$. In particular, on average, the eavesdropper knows $C_{eav}\cdot |\bar{U}|=\left(1-C_{s}\right)\cdot \left|\bar{U}\right|$ bits of $S_{s}$, which means that on average the remaining $C_{s}\left|\bar{U}\right|=nF_{g}$ bits of $S_{s}$ are unknown. As an example, assume a security parameter n=128 bits, a guard factor $F_{g}=1.1$, and the parameters suggested in the previous section: $n_{t}=2$ and f=8, with $C_{s}=0.73$, $p_{r}=0.18$. Then, L=236 bits, $\lceil n/C_{s}\rceil =176$ and the probability that the first step is repeated (i.e., $|\bar{U}|<176$) is lower than 0.2%:

(9) $$\sum_{i=0}^{175}\left(\begin{array}{c} 236\\ i \end{array}\right){\left(1-0.18\right)}^{i}0.18^{236-i}\;=\;0.0017.$$

## 6. Conclusions

There are two major challenges for protecting smart supply chains when RFID systems are used for situational awareness: to protect the shipment of goods (in particular, reduce inventory shrinkage and prevent unauthorized tracking), and to secure ownership transfer. In this paper, we have addressed both challenges. We have presented an anonymous scanning proof with missing tag identification that can be used to authorize untrusted carriers to track pallets of tagged goods, check their integrity, and identify any missing items without requiring a packing list. The authorization is for only one scanning, after which the goods are untraceable. For ownership transfer, backward privacy has been addressed using a novel approach based on positive secrecy channels with modified random-slotted modulation. This approach does not require TTPs or an IsE. An analysis of this channel is carried out, leading to optimal implementations with two noisy tags and eight frame slots.

## Appendix A

The value of the secrecy capacity (Equation (1)) can be obtained from the value of the following terms: $\left|X|,|Y|,p(x_{j},y_{k}\right)$ and $p(x_{j}|y_{k})$. To compute it, we shall consider three cases: (I) the general case when irreconcilable collisions are not removed, (II) the case when irreconcilable collisions are removed, and (III) the case when input data is also coded using the frame slot order. We shall assume that $2f\geq n_{t}+1$.

### Appendix A.1. General Case: |X|=2, with Irreconcilable Collisions

In this case, $p\left(x_{j}\right)=\frac{1}{2}$, j=0,1. Then,

(A1) $$|Y|\;=\;\left(\begin{array}{c} 2f+n_{t}\\ n_{t}+1 \end{array}\right)\;\;,$$

and, $p(y_{k})$, k=0,…,|Y|-1, is computed using a multinomial coefficient:

(A2) $$p\left(y_{k}\right)\;=\;{\left(\frac{1}{2f}\right)}^{n_{t}+1}\left(\begin{array}{c} n_{t}+1\\ Z_{1}^{0},Z_{2}^{0},\ldots ,Z_{f}^{0},Z_{2}^{1},Z_{2}^{1},\ldots ,Z_{f}^{1} \end{array}\right),\;\;\;k=0,\ldots ,\left|Y\right|-1,$$

where $Z_{i}^{0}$ and $Z_{i}^{1}$ are the numbers of tags that used slot *i* to respond with 0 and 1, respectively, so that $n_{t}=\sum_{i=1}^{f}\left(Z_{i}^{0}+Z_{i}^{1}\right)$.

Let $W_{k}^{0}=\sum_{i=1}^{f}\left(Z_{i}^{0}>0\right)$ and $W_{k}^{1}=\sum_{i=1}^{f}\left(Z_{i}^{1}>0\right)$. The joint probability $p(x_{j},y_{k})$ can be computed as: $p\left(x_{j},y_{k}\right)=p\left(x_{j}|y_{k}\right)\cdot p\left(y_{k}\right)$, where

(A3) $$p\left(x_{j}|y_{k}\right)\;=\;\frac{W_{k}^{j}}{W_{k}^{0}+W_{k}^{1}}\;\;,\;\;\;j=0,1,\;k=0,\ldots ,\left|Y^{\prime }\right|-1.$$

### Appendix A.2. |X|=2, without Irreconcilable Collisions

Let $S_{u}$ be the set of symbols of Y for which there are irreconcilable collisions. The new alphabet $Y^{\prime }$ is obtained by removing $S_{u}$ from Y: $Y^{\prime }=Y\setminus S_{u}$. The size of $Y^{\prime }$ can be computed as:

(A4) $$|Y^{\prime }|=\left(\begin{array}{c} 2f\\ n_{t}+1 \end{array}\right).$$

The probability $p(y_{k}^{\prime })$ of a new output symbol is: $p\left(y_{k}^{\prime }\right)=p\left(y_{k}\right)\cdot p_{s}^{-1}$, where $p_{s}$ is the probability of an output symbol not having an irreconcilable collision. The probability $p_{s}$ is the complement of the sum of the probabilities of the symbols in $S_{u}$:

(A5) $$p_{s}\;=\;1-\sum_{y_{k}\in S_{u}}y_{k}\;\;=\;\;\frac{2f!}{\;{\left(2f\right)}^{n_{t}+1}\left(2f-n_{t}-1\right)!}=\;\;\frac{(n_{t}+1)!}{\;{\left(2f\right)}^{n_{t}+1}}\left(\begin{array}{c} 2f\\ 2f-n_{t}-1 \end{array}\right)\;.$$

In this case, when applying the binomial coefficient (Equation (A2)), $Z_{i}^{0}$ and $Z_{i}^{1}$ are always either 0 or 1, so that the $y_{k}^{\prime }$ are equiprobable:

(A6) $$p\left(y_{k}^{\prime }\right)\;=\;{\left(\frac{1}{2f}\right)}^{n_{t}+1}\frac{(n_{t}+1)!}{p_{s}}\;\;=\;\;{\left(\begin{array}{c} 2f\\ 2f-n_{t}-1 \end{array}\right)}^{-1},\;\;\;\;k=0,\ldots ,\left|Y^{\prime }\right|-1.$$

Finally, the conditional probability $p(x_{j}|y_{k}^{\prime })$ is:

(A7) $$p\left(x_{j}|y_{k}^{\prime }\right)\;=\;\frac{W_{k}^{j}}{n_{t}+1}\;,\;\;\;j=0,1,\;k=0,\ldots ,\left|Y^{\prime }\right|-1.$$

### Appendix A.3. |X|=2f, without Irreconcilable Collisions

Input data are coded using the slot order and bit value (0 or 1), so that $x_{j}$, j=0,…,f-1, correspond with 0 in the slot j+1, while $x_{j}$, j=f,…,2f-1, is coded responding with 1 in the slot j+1-f. Thus, |X|=2f, $H\left(X\right)=\log_{2}\left(2f\right)$ and $p\left(x_{j}\right)=\frac{1}{2f}$, j=0,…,2f-1. The output alphabet is still $Y^{\prime }$, and the value of $p(y_{k}^{\prime })$ is the same (Equation (A6)). The value $p(x_{j}|y_{k}^{\prime })$ changes: $p(x_{j}|y_{k}^{\prime })$, j=0,…,f-1, is now 0 for those $y_{k}^{\prime }$ with $Z_{j+1}^{0}=0$, and $1/(n_{t}+1)$ otherwise. Likewise, $p(x_{j}|y_{k}^{\prime })$, j=f,…,2f-1, is 0 for the $y_{k}^{\prime }$ with $Z_{j+1-f}^{1}=0$ and $1/(n_{t}+1)$ otherwise. Thus:

(A8) $$\sum_{k=0}^{|Y^{\prime }|-1}p\left(x_{j}|y_{k}^{\prime }\right)\log_{2}p\left(x_{j}|y_{k}^{\prime }\right)\;=\;\left(\begin{array}{c} 2f-1\\ n_{t} \end{array}\right)\;\frac{1}{n_{t}+1}\;\log_{2}\frac{1}{n_{t}+1}\;\;,$$

and a closed expression for the secrecy capacity can be obtained:

(A9) $$H\left(X|Y\right)\;=\;-\left|X\right|\sum_{k=0}^{|Y^{\prime }|-1}p\left(x_{j}|y_{k}^{\prime }\right)\log_{2}p\left(x_{j}|y_{k}^{\prime }\right)p\left(y_{k}^{\prime }\right)\;=\;\log_{2}\left(n_{t}+1\right).$$
